# Effect of silver ion and silicate group on the antibacterial and antifungal properties of nanosized hydroxyapatite

**DOI:** 10.1038/s41598-024-80303-7

**Published:** 2024-11-26

**Authors:** Nataliia D. Pinchuk, Agata Piecuch, Natalia Charczuk, Paulina Sobierajska, Sara Targonska, Oleksii Bezkrovnyi, Rafał Ogórek, Yadong Wang, Rafal J. Wiglusz

**Affiliations:** 1 Institute of Low Temperature and Structure Research, Polish Academy of Sciences, Okólna 2, Wrocław, 50-422 Poland; 2 Frantsevich Institute for Problems of Materials Science of the NAS of Ukraine, Pritsaka, 3, Kyiv, 03142 Ukraine; 3 Department of Mycology and Genetics, University of Wroclaw, Przybyszewskiego 63/77, Wroclaw, 51-148 Poland; 4 Department of Molecular Sciences, Swedish University of Agricultural Sciences, Box 7015, Uppsala, 75007 Sweden; 5 Meinig School of Biomedical Engineering, College of Engineering, Cornell University, Ithaca, NY 14853-1801 USA; 6 Department of Organic Chemistry, Bioorganic Chemistry and Biotechnology, Silesian University of Technology, Krzywoustego 4, Gliwice, 44-100 Poland

**Keywords:** Hydroxyapatite compounds, Silver, Silicate, Antibacterial properties, Antifungal properties, Infection, Biomaterials

## Abstract

Hydroxyapatite is one of the most widely used materials in biomedical applications in reparative and regenerative medicine. Doping of nanosized hydroxyapatite improves its bioactive properties, and thus, the synthesis of different types of nanohydroxyapatite with antimicrobial activity is a perspective route of modern materials science. In this study, undoped hydroxyapatite (HAp), hydroxyapatite doped with silver (HAp with 0.1, 0.5 and 1 mol% Ag^+^ ions), and silicate-substituted hydroxyapatite doped with silver (Si-HAp with 0.1, 0.5 and 1 mol% Ag^+^ ions) nanoparticles (NPs) were synthesized by microwave-assisted hydrothermal technique and sintered at 450 °C. The structural properties and composition of obtained hydroxyapatite NPs were investigated using X-ray powder diffraction (XRPD), Fourier-transformed infrared spectroscopy (FT-IR), and Energy-dispersive X-ray spectroscopy (EDS). The morphology of synthesized nanosized powders was detected using the high-resolution transmission electron microscopy (HRTEM) technique. The results of XRPD for all synthesized nanosized powders confirmed the presence of hydroxyapatite crystal structure. The FT-IR spectra confirmed the presence of functional groups characteristic of the hydroxyapatite structure. The EDS analysis of obtained materials has shown the presence of Ca, P, O, Si, and Ag elements. Significant differences in size and morphology of the obtained particles were found using HRTEM. The particles have an elongated, rod-like shape with subtle differences. Moreover, HAp doped with 1 mol% Ag^+^ ions and Si-HAp doped with 1 mol% Ag^+^ ions nanosized powders showed antibacterial activity in comparison to pure hydroxyapatite both against gram-positive and gram-negative bacterial strains (*Klebsiella pneumoniae*, *Pseudomonas aeruginosa*, *Staphylococcus epidermidis*, *Staphylococcus aureus*, *Enterococcus faecalis*). These two types of hydroxyapatite NPs also demonstrated antifungal activity against reference strains of *Candida albicans*,* Candida kruzei*, and *Candida tropicalis*, with stronger activity observed for Si-HAp doped with silver.

## Introduction

Minerals within the apatite group demonstrate widespread occurrence in natural environments and biological specimens^1^. The hydroxyapatite (HAp), with the formula Ca_10_(PO_4_)_6_(OH)_2_, is the primary inorganic component of vertebrate bones and teeth. Le Geros described bone mineral as a carbonate-hydroxyapatite, with the chemical formula (Ca, X)_10_(PO_4_, HPO_4_, CO_3_)_6_(OH, Y)_2_, where X^n+^ represents cations that can replace calcium ions (Mg^2+^, Na^+^, Sr^2+^), and Y^−^ represents anions that can substitute for the hydroxyl group (Cl^−^ or F^−^)^1^. Gross and Berndt further reported that in addition to these elements, natural apatite (found in bone, enamel, and dentine) contains trace amounts of various ions, including K^+^, Ba^2+^, Pb^2+^, Fe^3+^, Zn^2+^, Cu^2+^, Al^3+^, Si^4+^, Mn^2+^, Se^2+^, Sn^2+^, K^+^, Li^+^, Ni^2+^, Ag^+^ are present in trace quantities within natural apatite (found in bone, enamel, and dentine)^2^. Synthetic high-aspect-ratio nanostructured HAp is attractive for a wide range of applications such as replacement bone tissue, drug delivery systems, catalysis, and environmental, and can interact with biological systems, such as cells and proteins, in unique ways^3^.

In this study, we focus on incorporating silicon and silver ions in synthetic HAp due to their distinctive and potentially beneficial properties. Silicon plays an important role in bone and cartilage systems^4^. Incorporating 0.8 and 1.5 wt% of orthosilicate groups into hydroxyapatite has been shown to significantly increase the rate of bone apposition to HAp bioceramic implants after 6 and 12 weeks. The mechanism by which silicate ions increase in vivo bioactivity has been explained, highlighting the enhanced potential of these ceramics for biomedical applications^5^. Authors of other work^6^ demonstrated the effect of silicon-substituted HAp on the differentiation of human osteoblasts and their ability to induce mineralization compared to pure HAp. Silicate anions (SiO_4_^4−^) can structurally be included in the HAp lattice instead of phosphate ions. This substitution affects the crystal lattice parameters of silicon-substituted HAp and depends on the content of silicon groups, the synthesis method, and the subsequent heat-treatment temperature. Authors of other studies^7,8^ have shown that (PO_4_)^3−^ groups could be substituted for (SiO_4_)^4−^ in the silicon-substituted HAp without forming other crystalline phases. Substitution of phosphate group by silicate in the apatite structure increases the lattice parameters in both a-axis and c-axis of the unit cell^9^. Silicon-substituted HAp can be prepared by high-temperature solid-state reaction using Ca_2_P_2_O_7_, CaCO_3,_ and SiO_2_^10^ by hydrothermal method using tetraethylorthosilicate Si(OCH_2_CH_3_)_4_ (TEOS) solution^11^ or tetramethylammonium silicate (CH_3_)_4_N(OH)·2H_2_O (TMAS)^12^, by precipitation method^13–15^ and others. However, when using silicon acetate (Si(CH_3_COO)_4_ in a precipitation method together with high sintering temperature it can lead to the formation of biphasic or multiphase calcium phosphate ceramic^16^. By using microwave heating, Si-HAp nanocrystals could be nucleated rapidly with high efficiency compared to other methods^17^. Additionally, a microwave-assisted hydrothermal method can contribute to producing silicate-substituted HAp nanoparticles (HAp NPs) doped with various ions^18^. It can be rare-earth ions, for example, Eu^3+^ ions^19,20^, which have good luminescent properties. In this case, the doping ion’s choice depends on the synthesized material’s purpose.

It is well established that any medical procedure or intervention in the human body carries a risk of infections. Therefore, the use of antibiotics is an integral part of the treatment protocol for medical interventions. Due to the increased drug resistance among microorganisms observed in recent years, it is crucial to search for novel strategies to prevent and treat microbial infections. Doping hydroxyapatites with various ions can change their properties, for instance Sr enhance HAp solubility and Mg increase its fracture toughness. For antibacterial activity Zn^2+^ and Ag^+^ dopants have been chosen most frequently, with the greater growth inhibition observed for Ag-doped HAp^21^). The antibacterial properties of silver are well-known. Moreover, the development of multi-resistant bacteria, as in the case of antibiotics, is less likely^22^. This effect is caused mainly by silver ions, which have a wide antibacterial spectrum, and also by silver nanoparticles (Ag NPs)^22^. In addition, some studies show the presence of a synergistic effect of some antibiotics and silver^23^. Hydroxyapatite doped with silver has been synthesized using methods such as sol-gel^24,25^, precipitation^26–29^, hydrothermal synthesis^30^, and microwave-assisted techniques^31^. It had indicated that incorporating silver ions into HAp could contribute viably to excellent antibacterial activities against *P. aeruginosa* to prevent bone infection^32^. It is crucial to select the optimal concentration of silver for doping hydroxyapatite to minimize silver toxicity. The cell adhesion assays showed that osteoblast cell attachment in varying density was noticed on HAp samples doped with 0.5, 1 and 1.5 mol% Ag^+^ ions. However, osteoblast function was significantly greater on HAp 0.5 mol% Ag^+^ compound now than on 1 and 1.5 mol% Ag^+^ ions samples, and osteoblast cells were attached but could not spread as HAp doped with 0.5 mol% Ag^+^ ions suggesting that HAp compounds with a low amount of silver are more favorable for implant applications^31^. It is important to highlight the varied cellular responses and cytotoxic effects induced by AgNPs and HAp NPs. Previous studies^33^have described the initial cellular response to AgNP exposure, suggesting mechanisms such as apoptosis or necrosis, particularly at concentrations of 50 µg mL⁻¹, which also resulted in decreased cell adhesion. In contrast, HAp NPs showed a potential for recovery in cell proliferation, even at higher concentrations, indicating a more biocompatible interaction with cells. HAp doped with silver ions demonstrates a clear antimicrobial effect^26–29,32^, including applications in theranostics^27^. Considering that the silicate group in apatite improves its bioactive properties and silver ions have an antibacterial effect, combining these substitutions in one material would be interesting and promising. Our previous work demonstrated antifungal Ag-doped Si-HAp NPs synthesized via the hydrothermal method and sintered at 600 ºC in which three phosphate groups ((PO_4_)^3−^) were replaced with three silicate groups ((SiO_4_)^4−^)^30^. It is crucial to investigate whether reducing the amount of dopant and lowering the sintering temperature can preserve the antimicrobial and antifungal properties of doped hydroxyapatite, and how these modifications compare to undoped hydroxyapatite.

The present study aims to prepare silver-doped hydroxyapatite and silver-doped silicate-substituted hydroxyapatite nanoparticles and characterize them in comparison to undoped hydroxyapatite nanoparticles, including X-ray powder diffraction, energy dispersive X-ray spectroscopy, Fourier-transform infrared spectroscopy, and high-resolution transmission electron microscopy, as well as its antibacterial and antifungal properties. Such nanoparticles could be used as medical materials, enhancing biocompatibility and reducing the risk of microbial infections during implantation procedures.

## Materials and methods

### Synthesis of the undoped hydroxyapatite, silver-doped hydroxyapatite, and silver-doped silicate-substituted hydroxyapatite NPs

#### Synthesis of the undoped hydroxyapatite NPs (HAp NPs)

As substrates, the following were used: Ca(NO_3_)_2_·4H_2_O (99.0–103.0%, Alfa Aesar, Haverhill, MA, USA), (NH_4_)_2_HPO_4_ (> 99.0%, Acros Organics, Schwerte, Germany). To obtain stoichiometric hydroxyapatite, the concentrations of reagents were selected according to the required Ca/P molar ratio of 1.67. Stoichiometric amounts of substrates were dissolved separately in deionization water. All starting substrates were mixed into a Teflon vessel. The ammonia solution (NH_3_·H_2_O, 25%, Avantor, Poland) was used to obtain a pH level of around 9–10. The hydrothermal synthesis was conducted in a microwave reactor (ERTEC MV 02–02, Wrocław, Poland) for 90 min at 220–250 °С and under autogenous pressure (42–45 bar)^20^. The obtained material was dried at the temperature of 80 °С and then heat-treated at the temperature of 450 °C for 3 h (3 °C/min).

#### Synthesis of the silver-doped hydroxyapatite NPs (Ag-doped HAp NPs)

The synthesis of silver-doped hydroxyapatite was carried out as described above in paragraph 2.1.1. The source of silver ions was AgNO_3_ (99.9% Avantor Performance Materials Poland S.A, Gliwice, Poland). The chosen concentrations of silver were 0.1, 0.5, and 1 mol% in a ratio of calcium ion molar content. Obtained NPs were named HAp: 0.1 mol% Ag^+^, HAp: 0.5 mol% Ag^+^, and HAp: 1 mol%Ag^+^.

#### Synthesis of the silver-doped silicate-substituted hydroxyapatite NPs (Ag-doped Si-HAp NPs)

The synthesis of silver-doped silicate-substituted hydroxyapatite was carried out as described above in paragraph 2.1.1. The source of silver ions was AgNO_3_ (99.9% Avantor Performance Materials Poland S.A, Gliwice, Poland). The chosen concentrations of silver were 0.1, 0.5, and 1 mol% in a ratio of calcium ion molar content. The source of one silicate group was tetraethyl orthosilicate TEOS (> 99%, Alfa Aesar, Haverhill, MA, USA). Obtained NPs were named Si-HAp: 0.1 mol% Ag^+^, Si-HAp: 0.5 mol% Ag^+^ and Si-HAp: 1 mol% Ag^+^ .

### Characterization

#### X-ray powder diffraction (XRPD)

PANalytical X’Pert Pro X-ray diffractometer (Malvern Panalytical Ltd., Malvern, UK) with a Cu–K radiation at 2θ range from 10̣º to 80º (exposure time of 1,5 h) was applied to determine structure and crystallinity of obtained materials. The diffraction patterns were juxtaposed with standards from the Inorganic Crystal Structure Database (hydroxyapatite ICSD-151941 and silicate-substituted hydroxyapatite ICSD-32357).

The phase purity, average crystallite size and cell parameters were calculated by the Rietveld method. The structural refinement was performed using the Maud program version 2.55, based on the hexagonal apatite crystal structure with improved approximation and indexing of the Crystallographic Information File (CIF). The Levenberg–Marquardt algorithm was applied along with the pseudo-Voigt approximation. The quality of the structural refinement was evaluated using R_w_-values.

#### Energy dispersive X-ray spectroscopy (EDS)

The chemical composition was checked by an FEI Nova NanoSEM 230 scanning electron microscope equipped with an energy-dispersive spectrometer (EDAX Genesis XM4). The EDS analysis has shown the existence of Ca, P, O, Si, and Ag elements in the obtained materials. The EDS spectra were recorded in triplicate for each sample, and the reported values represent the mean of these measurements.

#### Fourier transform infrared (FT-IR) spectroscopy

By FT-IR spectroscopy, the characteristic molecular vibrations were revealed. Spectra were collected by a Thermo Scientific Nicolet iS50 FT-IR spectrometer equipped with an Automated Beamsplitter exchange system (iS50 ABX containing DLaTGS KBr detector), built-in all-reflective diamond ATR module (iS50 ATR), Thermo Scientific Polaris™. The HeNe laser was used as an infrared radiation source. The FT-IR spectra were recorded using the ATR module, in the range of mid-infrared radiation.

#### High-resolution transmission electron microscopy

The morphology of the samples was determined by high-resolution transmission electron microscopy (HRTEM), using a Philips CM-20 SuperTwin instrument operating at 160 kV. Specimens were prepared by dispersing the sample in methanol and putting a droplet of the suspension on a microscope copper grid covered with carbon. Samples were then dried and purified in oxygen/hydrogen plasma in a plasma cleaner. Statistical analysis of TEM measurements data were done by grain size distribution curve.

### Antimicrobial activity of nanoapatites

Antibacterial and antifungal activity of silver-doped nanosized apatite compounds were tested on the following bacterial: *Staphylococcus aureus* ATCC 6538, *Staphylococcus epidermidis* ATCC 12,228, *Enterococcus faecalis* ATCC 51,299, *Klebsiella pneumoniae* subsp. *pneumoniae* ATCC 700,603, *Pseudomonas aeruginosa* ATCC 27,853 and fungal: *Candida albicans* ATCC 90,028, *Candida kruzei* ATCC 2159 and *Candida tropicalis* ATCC 750 strains. Colloidal solutions of nanosized apatite materials were prepared as described before^30^. Briefly, 10 mg/ml of HAp, HAp: 1 mol%Ag^+^ and Si-HAp: 1 mol%Ag^+^ in minimal medium (SD - Synthetic Defined for antifungal testing: Yeast Nitrogen Base 6.7 g/l, Glucose 20 g/l; Minimal Davies for antibacterial testing, ammonium sulfate 1 g/l, monopotassium phosphate 2 g/l, dipotassium phosphate 7 g/l, sodium citrate 0.5 g/l, magnesium sulfate 0.1 g/l, glucose 1 g/l) was sonicated and diluted 10x in an appropriate medium. Antibacterial activity was tested in a 96-well flat-bottom microtiter plate. Bacterial overnight cultures in LB medium (Luria Broth, Bioshop) were centrifuged and suspended in minimal medium to obtain optical density of 0.5 McF and diluted 10x in minimal medium. The wells were filled with 10 µl of bacterial solution, 10 µl of HAp, and 80 µl of the medium. Plates were incubated for 18 h at 37 ± 0.5 °C without shaking, and optical density was measured at 600 nm using a microplate reader (VarioSkan LUX, Thermo Fisher Scientific). Antifungal activity was measured in SD medium as described before^30^.

## Results and discussion

### X-ray powder diffraction analysis

**Fig. 1:**
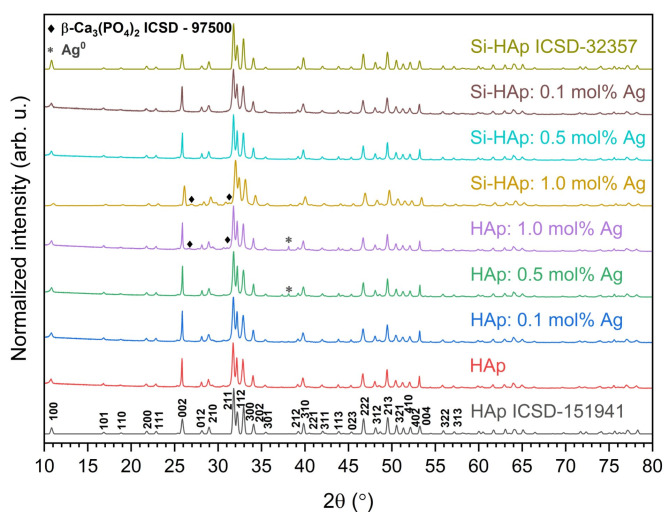
XRPD patterns of undoped HAp, HAp, and Si-HAp NPs doped with 0.1, 0.5, and 1.0 mol% Ag^+^ ions synthesized under hydrothermal conditions (at 240 °C and under 42–45 bar) and sintered at 450 °C.

The XRPD patterns of synthesized hydroxyapatite NPs have been shown in Fig. 1. It is known that hydroxyapatite crystallizes in hexagonal *P6*_*3*_*/m* space group with lattice constants an equal to 0.942 and *c* equal to 0.688 nm^34^. Obtained XRPD patterns of undoped HAp, HAp doped with silver, and Si-HAp doped with silver were compared with the X-ray pattern of hexagonal hydroxyapatite (ICSD-151941^10^), and silicate-substituted hydroxyapatite (ICSD-32357^35^), from the ICSD (Inorganic Crystal Structure Database), respectively. After sintering at 450 °С, the crystalline phase of hydroxyapatite was obtained in the samples of all compositions, but there are some differences between pure hydroxyapatite and both silver-doped HAp and silver-doped silicate-substituted HAp.

Early in work^27^ was established in the case of nanosized HAp doped with 1–2 mol% Ag^+^ ions (synthesized by precipitation method and sintered at 450 ºC), the sample HAp doped with 1 mol% Ag^+^ ions was monophasic, but the sample doped with 2 mol% Ag^+^ ions contained an additional phase of Ag^0^ (ICSD-22434^36^). It was also established that heat-treating of nanosized HAp doped only 0.5 mol% Ag^+^ ions (synthesized by microwave process) higher than 700 ºC leads to the presence of the additional phase of Ag^0 31^. Synthes HAp doped with mol% Ag^+^ ions *via* ultrasonic coupled sol-gel techniques with next heat treatment at 600 ºC also leads to the formation of secondary phases, including tricalcium phosphate phase (*β-*TCP)^37^. Now, in this work, the analysis of X-ray patterns of HAp doping is 0.1, 0.5, and 1 mol% Ag^+^ ions synthesized using a hydrothermal method with a microwave reactor that showed the presence of a small peak of metallic silver (ICSD-22434^36^) at about 2*θ* equal to ~ 38º for HAp doped with 0.5 and 1 mol% Ag^+^ ions, and this peak is more noticeable with increasing content of silver. Moreover, silicate substituted HAp doped with 0.1, 0.5 and 1 mol% Ag^+^ ions, the peak of silver, is not just as noticeable. This is most likely due to some changes in the crystal lattice when replacing phosphate ions with silicone ions in the hydroxyapatite structure, as was established earlier^12^, and this contributes to better incorporation of doped ions into the hydroxyapatite structure^20^.

**Fig. 2:**
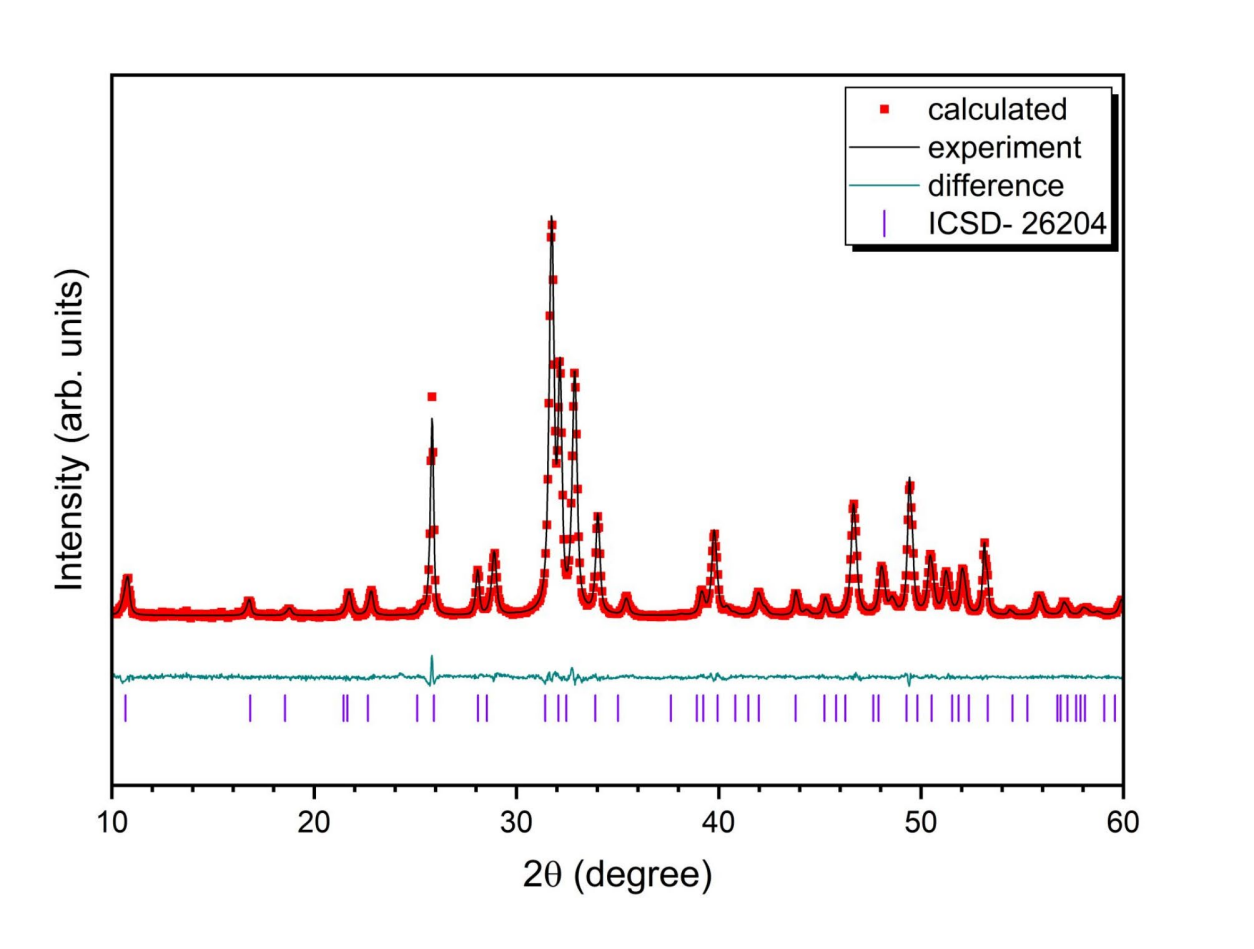
Representative Rietveld analysis results of the nanosized 0.1 mol% Si-HAp: Ag^+^, sintered at 450 °C, (black – experimental result; red – fitted diffraction; green – differential pattern, violet column – reference phase peak position).

The formation of *α-* or *β-*TCP phases during the sintering of hydroxyapatite could be observed in both cases: when the phosphate group is replaced by silicate group^13^ and when calcium ions are replaced by silver ions in the hydroxyapatite structure^31^. For instance, the *α-*TCP formation was observed with replacement higher than one phosphate group (1.25) in silicon-substituted hydroxyapatite synthesized by a precipitation route and heat-treated at 1000 °C^13^. Also, early *β-*TCP formation was observed in HAp doped with Ag^+^ ions ≥ 1.5 at. mol% synthesized by microwave process and heat-treated at 900 °C^31^. Thus, two types of substitution (anionic and cationic) and their combination, as well as the use of hydrothermal synthesis that promoted the preparation of nanosized powders, could contribute to the *β-*TCP formation. In general, the presence of a small amount of the *β-*TCP phase in the prepared materials is a positive point, as it is known to have not only osteoconductive but also osteoinductive effects^38^. In this work XRPD patterns of Si-HAp: 1% Ag^+^ andHAp: 1% Ag^+^, aswell as HAp: 0.5 mol% Ag^+^ demonstrated the presence of some small additional peaks that showed secondary phase formation identified as *β-*TCP (ICSD-97500). It is mostly related to the higher concentration of silver (1 mol%) in these samples (see Table 1). The formation of the hexagonal phase and the successful substitution of silver and silicate ions were confirmed. Figure 2 shows a good match between the observed XRPD pattern and the theoretical fit. Minimal differences in intensity, as represented by the line (Y_Obs_ − Y_Calc_), demonstrate the success of the Rietveld refinement. Further details regarding the Rietveld analysis are provided in Table 1.

**Table 1:** Unit cell parameters (a, c), crystal cell volume (V), grain size (nm), as well as a refined factor (R_w_) for the HAp and *β*-TCP.

Cell parameters							Phase			
	HAp			β-TCP			HAp (%)	β-TCP (%)	grain size [nm]	*R*_w_ (%)
Sample	a (Å)	c (Å)	V (Å^3^)	a (Å)	c (Å)	V (Å^3^)				
s. c.	9.424(4)	6.879(4)	529.09(54)	10.4352(2)	37.4029(5)	3527.26(14)	**–**	**–**	**–**	**–**
HAp	9.429(3)	6.886(4)	530.25(13)	–	–	–	100	**–**	66.2(2)	4.0(4)
HAp: 0.1 mol% Ag^+^	9.430(9)	6.886(1)	530.40(82)	–	–	–	100	**–**	56.2(1)	3.9(3)
HAp: 0.5 mol% Ag^+^	9.430(1)	6.886(4)	530.34(13)	10.4358(6)	37.4013(0)	3527.55(52)	99.6	0.4	93.4(1)	4.6(3)
HAp: 1 mol% Ag^+^	9.432(2)	6.887(0)	530.62(38)	10.4393(0)	37.4023(9)	3529.98(40)	97.7	2.3	95.2(1)	4.5(1)
Si-HAp: 0.1 mol% Ag^+^	9.427(5)	6.889(3)	530.27(21)	–	–	–	100	**–**	47.7(2)	2.5(3)
Si-HAp: 0.5 mol% Ag^+^	9.429(1)	6.886(8)	530.25(97)	–	–	–	100	**–**	70.2(4)	2.9(1)
Si-HAp: 1 mol% Ag^+^	9.437(1)	6.891(0)	531.48(38)	10.4696(4)	38.0694(5)	3613.85(51)	94.8	5.2	43.4(2)	4.8(2)

s. c. – single crystal reference data, HAp – ICSD-26,204, *β*-TCP – ICSD-97,500^39^.

The cell parameters as well as grain average size were calculated. The results are collected in Table 1. When comparing the cell volume of undoped HAp with other materials after the addition of silver, we can see that these values are similar, but the highest one is seen for the Si-HAp: 1 mol% Ag. The average grain size is smaller for the silicate-substituted HAp with the same concentration of silver ions.

### Elemental analysis

**Fig. 3:**
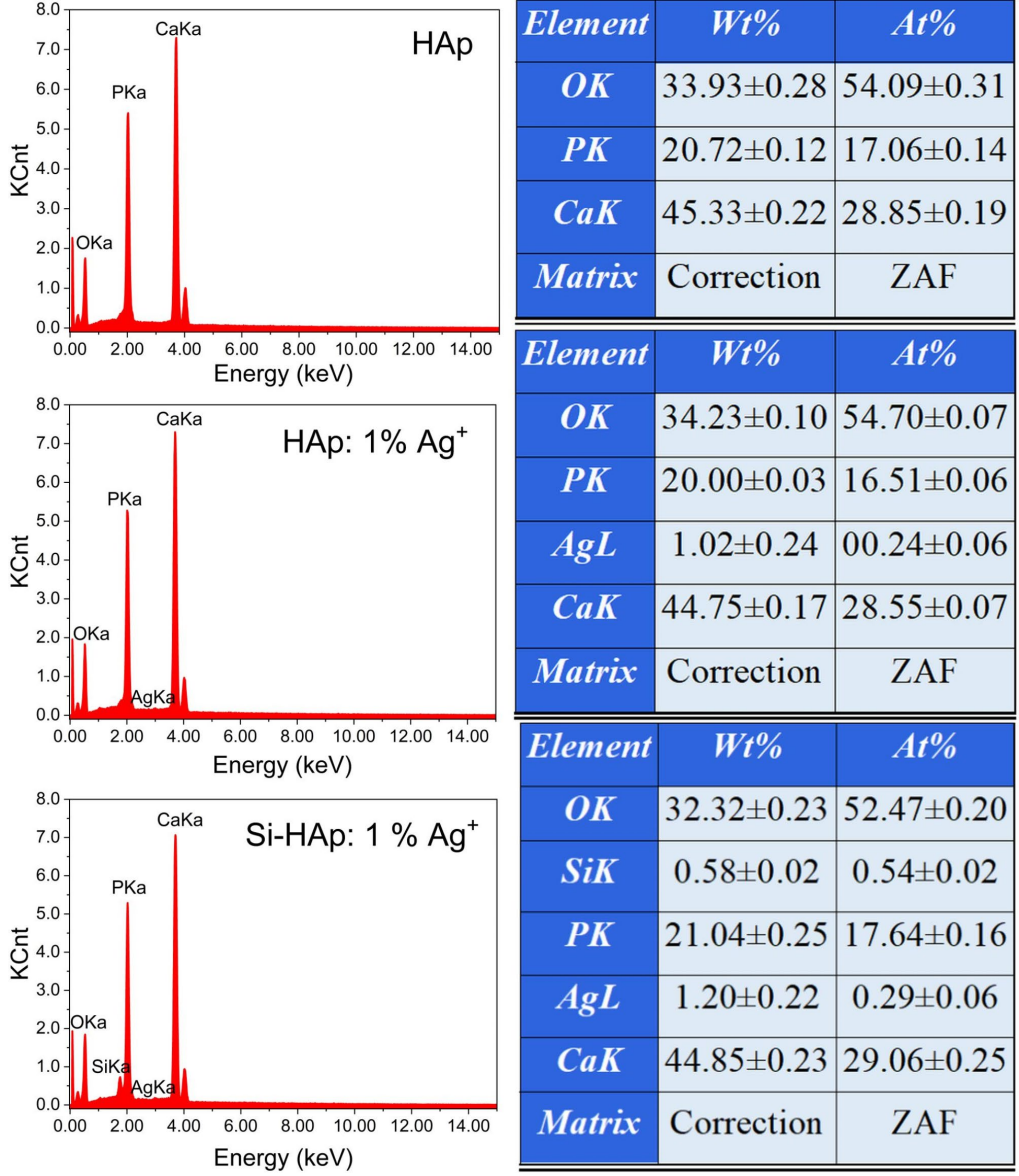
EDX analysis of undoped HAp, HAp: 1 mol% Ag^+^ and Si-HAp: 1 mol% Ag^+^, synthesized under hydrothermal conditions (at 240 °C and under 42–45 bar) and sintered at 450 °C.

The EDX spectra confirmed the presence of all elements (calcium, phosphorus, silver, silicon, and oxygen) in the prepared samples. Figure 3 shows the results of EDX analysis for undoped HAp, HAp doped with silver, and Si-HAp doped with silver (with the maximum amount of silver dopant − 1 mol%). Based on EDS analysis (Fig. 3, b, c), the concentrations of silver ions in HAp: 1 mol% Ag and Si-HAp: 1 mol% Ag samples are 0.97 mol% and 1.18 mol%, respectively, corresponding with the assumed value (1%). Regarding Si (Fig. 3, c), the content of this element equals 3.16 mol% (or 0.19 mol concerning the total mol number (6) of Si and P).

It is known that the molar ratio of Ca/P in stoichiometric hydroxyapatite with the formula Ca_10_(PO_4_)_6_(OH)_2_ is 1.67. However, the biological hydroxyapatite is non-stoichiometric due to the presence of other ions and functional groups^40^, and in the human body, the molar ratio also depends on the gender, age, and health condition of a patient. In the case of synthetic hydroxyapatite, the molar ratio of Ca/P depends on the method and parameters of synthesis and other conditions. In this work, the obtained value for pure HAp was 1.68 and practically corresponded to stoichiometric HAp. In the case of HAp doped with silver, the molar ratio (Ca + Ag)/P slightly deviated from such for pure HAp and was 1.75, and for silicate-substituted hydroxyapatite, the molar ratio (Ca + Ag)/(P + Si) was 1.57. The analogical molar ratio (Ca)/(P + Si) also was obtained in work^12^, where this ratio was 1.58 for sample Si-HAp with 0.36 (SiO_4_)^4−^ group (where the sum of (SiO_4_)^4−^ and (PO_4_)^3−^ equals 6) synthesized hydrothermal method. This correlates with our results since we obtained Ag^+^: Si-HAp, which also included less than one silicate group (Fig. 3).

### FT-IR analysis

**Fig. 4:**
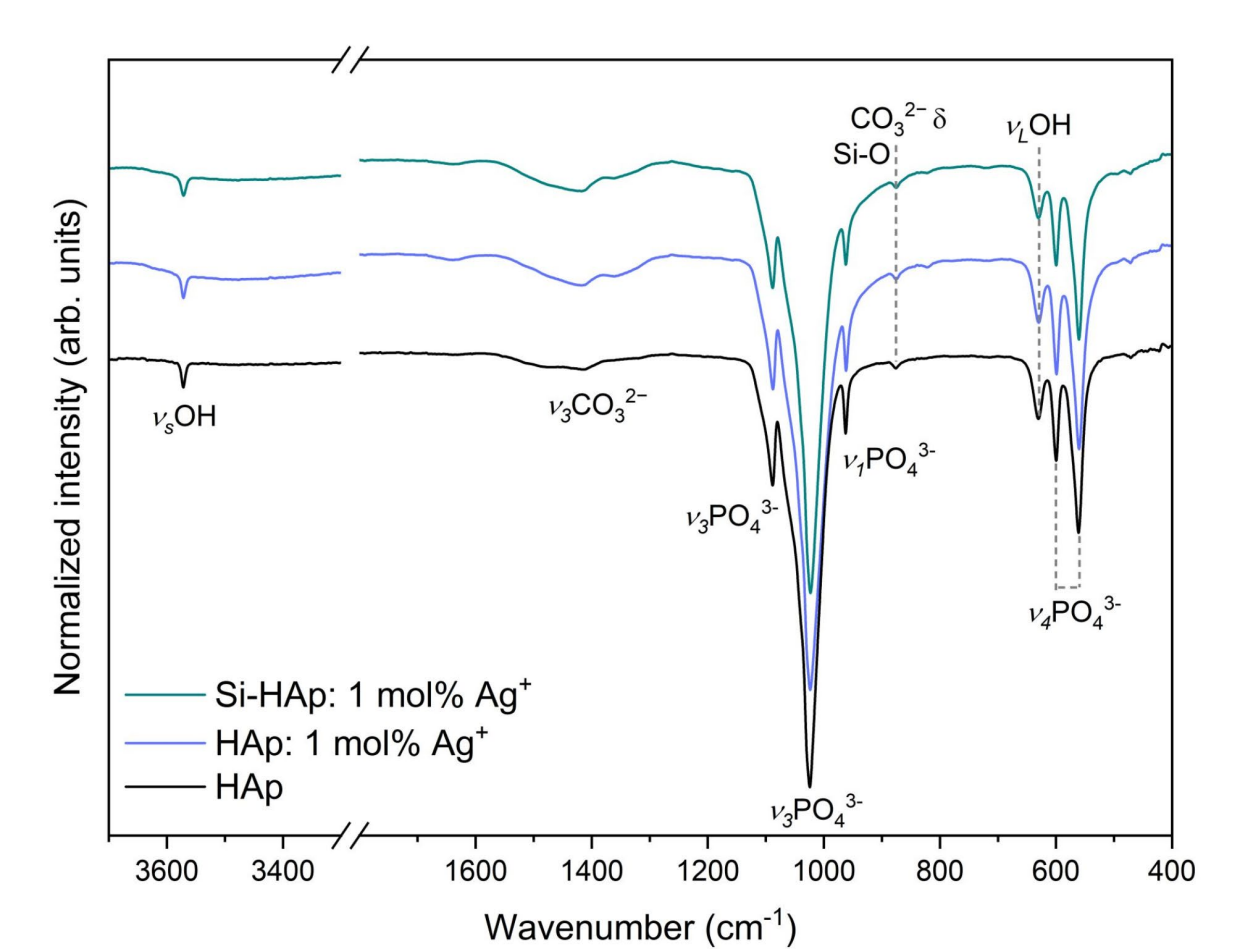
FT-IR spectra of undoped HAp, HAp: 1 mol% Ag^+^ and Si-HAp: 1 mol% Ag^+^, synthesized under hydrothermal conditions (at 240 °C and under 42–45 bar) and sintered at 450 °C.

Figure 4 shows FT-IR spectra of undoped HAp, HAp: 1 mol% Ag^+^, and Si-HAp: 1 mol% Ag^+^, sintered at 450 °C. Spectrum of undoped HAp shows bands corresponding to the vibrational modes of phosphate and hydroxide groups, characteristic of HAp^13^. The bands at 1100, 1034, and 962 cm^− 1^ (P-O stretching vibrations) and the doublet at 602 and 562 cm^− 1^ (O-P-O bending vibrations) originate from phosphate groups^17^. OH^−^ groups can be identified by the bands corresponding to stretching vibrations at 3572 cm^− 1^ and bending vibrations at 631 cm^− 1 17^. The same bands are present in the spectra of the samples HAp: 1 mol% Ag^+^ and Si-HAp: 1 mol% Ag^+ 17,20^, with no significant difference in the shapes and intensity of the peaks compared to the spectra of undoped HAp.

The visible broad band in the 1400–1600 cm^− 1^ region originates from carbonate ions (CO_3_^2−^). The maximum is visible at 1415 cm^− 1^ (ν₃, asymmetric stretching vibrations). This band is present in all spectra (undoped HAp, HAp: 1 mol% Ag^+^, and Si-HAp: 1 mol% Ag^+^). The presence of carbonates is due to “B-type” carbonate substitution when carbonate ions substitute for phosphate^41^. The reason behind the spontaneous carbonation is the adsorption of CO_2_ from the air during the synthesis and calcination. Additionally, the band detected at 875 cm^− 1^ originates from the bending vibrations of the carbonate group (δCO_3_^2−^), also related to intrinsic carbonation^42^, as well as it is overlaid with the vibrational modes from Si-O^20^. This band may also correspond to the stretching vibrations of hydrogen phosphate ions (ν_s_HPO_4_^2−^), which could result from calcium deficiency linked to the first charge compensation mechanism. However, as the band assigned to the carbonates is also detected in the 1400–1600 cm^− 1^ region, the hydrogen phosphate origin is a secondary consideration.

Some decrease of 631 cm^− 1^ and 3572 cm^− 1^ modes for the OH^−^ group due to the presence of the (SiO_4_)^4−^ group, which coincides with the data for silicate-substituted apatite^17^. According to the authors^43^, these changes in the hydroxyl stretching bands at 3570 and 630 cm^− 1^ are the most notable effects of ionic substitution on comparing the IR spectra of the pure and Si-substituted hydroxyapatite because the substitution of silicon into the hydroxyapatite framework reduces the amount of hydroxyl group to compensate for the extra negative charge of the silicate group and confirmed (PO_4_)^3−^ tetrahedra replacement by (SiO_4_)^4−^ tetrahedra in the hydroxyapatite structure.

### High-resolution transmission Electron Microscopy

**Fig. 5:**
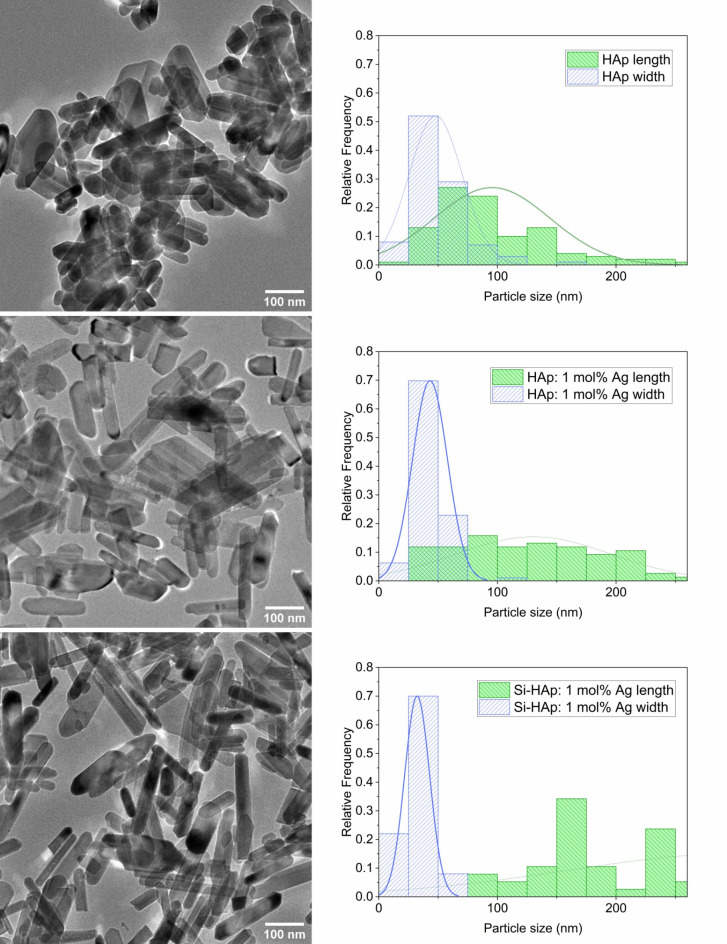
TEM images and particle size distribution of undoped HAp NPs, HAp: 1 mol% Ag^+^ NPs, and Si-HAp: 1 mol% Ag^+^ NPs synthesized under hydrothermal conditions (at 240 °С and under 42–45 bar) and sintered at 450 °C.

The study of the morphology of undoped and doped HAp NPs is of great importance, as the shape and size of the particles can significantly influence their properties. Figure 5 shows TEM images and the particle size distribution of undoped HAp, HAp: 1 mol% Ag^+^, and Si-HAp: 1 mol% Ag^+^ nanosized particles sintered at 450 °C. Moreover, the grain size distribution curve has been presented in Fig. 5.

From the TEM images, it can be seen that the crystals of undoped HAp NPs synthesized under microwave hydrothermal conditions have an elongated rounded shape, namely nanorods, and wide size distribution. The HAp particle size is in the wide range of 20–260 nm in length and 10–160 nm in width. The analysis of particle size distribution showed the presence of the main fraction 40–60 nm for length and width with the maximum peak in the range 20–40 nm. Generally, the average particle size in length is 96 nm, and in width is 48 nm.

Nanocrystals of HAp: 1 mol% Ag^+^ have a more elongated shape than pure HAp crystals and narrower particle length distribution. Particle size distribution is in the range of 20–300 nm in length with a maximum peak in the range of 80–100 nm and 20–120 nm in width with a maximum peak of 20–40 nm. Thus, adding silver to HAp leads to the formation of longer particles with an average length size of 130 nm but thinner ones with an average width size of 43 nm.

**Fig. 6:**
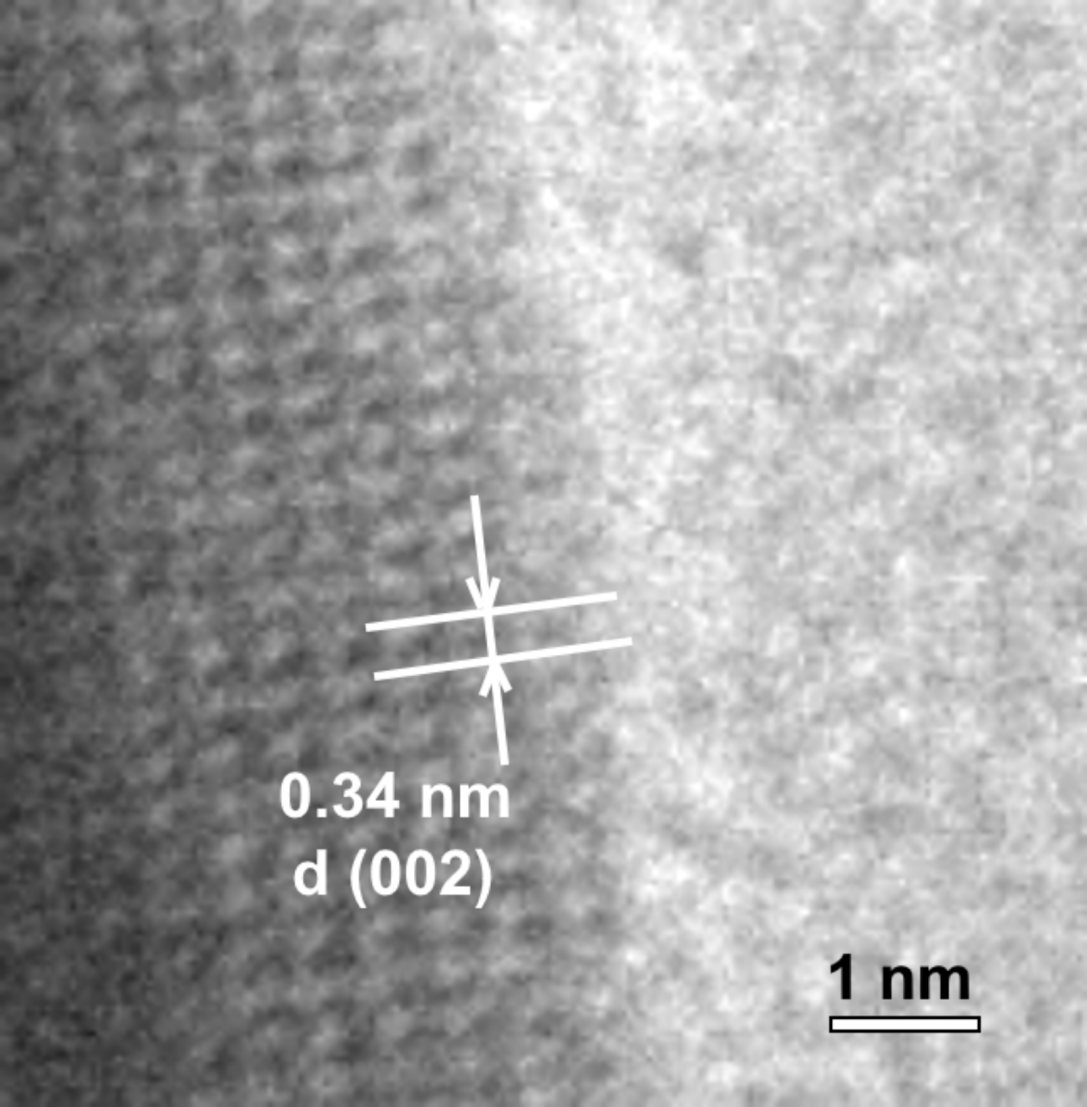
High-resolution lattice image of Si-HAp: 1 mol% Ag^+^ NPs synthesized under hydrothermal conditions (at 240 °С and under 42–45 bar) and sintered at 450 °C.

Particles of Si-HAp: 1 mol% Ag^+^ have an even more elongated shape than undoped HAp and HAp: 1 mol% Ag^+^ particles, which are intermediate between rods and needles. Replacing a group (PO_4_)^3−^ with a group (SiO_4_)^4−^ in the sample HAp: 1 mol% Ag^+^ contributes to a significant decrease in particle in length (average size of 124 nm) and in width (average size of 32 nm). Particle size distribution is in the range of 25–275 nm in length (a maximum peak is in the range of 80–100 nm) and 10–70 nm in width (a maximum peak is in the range of 25–50 nm). A high-resolution lattice image of Si-HAp: 1 mol% Ag^+^ NPs is presented in Fig. 6.

In general, for both undoped and doped HAp NPs, the width particle size maximum peaks in the 25–50 nm range, while for the length particle size, the peak is 50–75 nm for undoped HAp and shifts to larger sizes for doped HAp. Ag-HAp NPs made by modified sol–gel technique was shown to have elongated rod-like morphology with the length of the nanorods varying from 110 to 180 nm and the diameter varying between 20 and 25 nm^25^.

Thus, it has been established that doping with silver and dual doping with silver and (SiO_4_)^4−^-group significantly affect the morphology of hydroxyapatite nanocrystals, which can subsequently affect the properties of prepared materials, including antibacterial and antifungal activities. HAp NPs doped with bioactive ions and synthesized by microwave-assisted hydrothermal technique exhibited a change in morphology in previous studies^44^. The size of Ce-substituted HAp NPs increased with the Ce^3+^ concentration, while in the case of Mg-substituted HAp NPs, a change in the morphology was observed as the Mg^2+^ concentration increased, acquiring a platelet shape at 5% substitution^44^.

### Antimicrobial activity of nanosized apatite compounds

#### Antibacterial activity

**Fig. 7:**
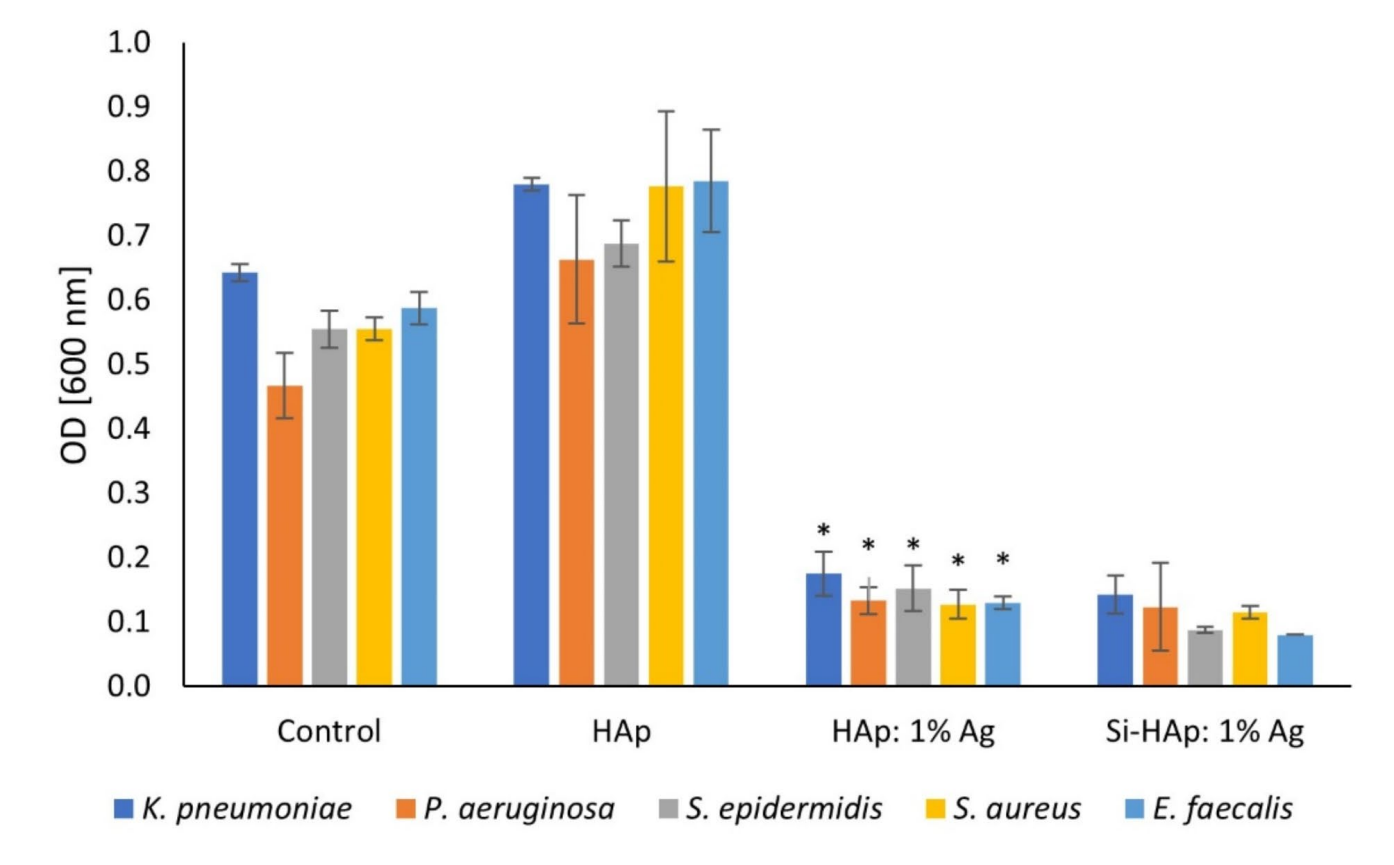
The inhibition of bacterial growth by the control group, undoped HAp, HAp: 1 mol% Ag^+^ and Si-HAp: 1 mol% Ag^+^ NPs against reference strains *Klebsiella pneumoniae*, *Pseudomonas aeruginosa*, *Staphylococcus epidermidius*, *Staphylococcus aureus*, and *Enterococcus faecalis*: mean ± SD, *n* = 3, *- statistically significant (*p* < 0.05).

Antibacterial effects of nanoapatites against gram-positive (*S. aureus*,* S. epidermidis* and *E. faecalis*) and gram-negative strains (*K. pneumoniae* and *P. aeruginosa*) are shown in Fig. 7. The growth of all tested strains was significantly inhibited at the concentration of 0.1 mg/ml of Ag^+^-HAp and Ag^+^-Si-HAp (*p* < 0.05). The antibacterial activity of silver in all its forms is well documented. Silver ions have multiple effects on bacterial cells, interacting with major enzymes and disrupting plasma membranes, among others^45^. As hydroxyapatites are applied as medical materials, developing novel structures to increase their biological properties is important. HAps doped with silver ions were shown to have antimicrobial potential against gram-positive and gram-negative bacteria. For instance, Ag-doped HAp developed by Ciobanu et al. was active against *S. aureus* and *K. pneumoniae*^46^. Ivankovic et al. showed that Ag^+^ ions substituted hydroxyapatite exhibited antimicrobial activity against *Acinetobacter baumanii* compared to pure HAp^47^. We compared the antibacterial activity of silver-doped HAp modified and unmodified with silica. It was shown that silica-substituted Ag^+^-HAp has a slightly better antibacterial effect against *S. epidermidis* and *E. faecalis*. However, the activity against the remaining strains was similar (Fig. 7). We did not observe any significant difference in the antibacterial activity of Ag^+^-doped HAps against gram-positive and gram-negative strains, consistent with some published data. Ciobanu et al. showed similar inhibitory effects of Ag^+^: HAp NPs against *S. aureus* and *K. pneumonia* On the other hand, HAp modified with silver nanosized particles exhibited much stronger antibacterial activity against gram-negative strains than gram-positive ones^48^. Gram-positive bacteria possess thick cell walls that may prevent nanoparticles from penetratingthe plasma membrane^45^. Since one the main medical applications of nanoapatite is bone replacement, implanted material should be modified in a way to reduce the risk of post-surgical infection. Osteomyelitis is one of the most common complications of orthopedic surgery, with *S. aureus*, *S. epidermidis* and *P. aeruginosa* as most frequent etiological agents^49^. Silica-substituted Ag^+^-HAps developed by our team show strong activity against these microbes, thus they are promising orthopedic materials that inhibit the risk of infection.

#### Antifungal activity

**Fig. 8:**
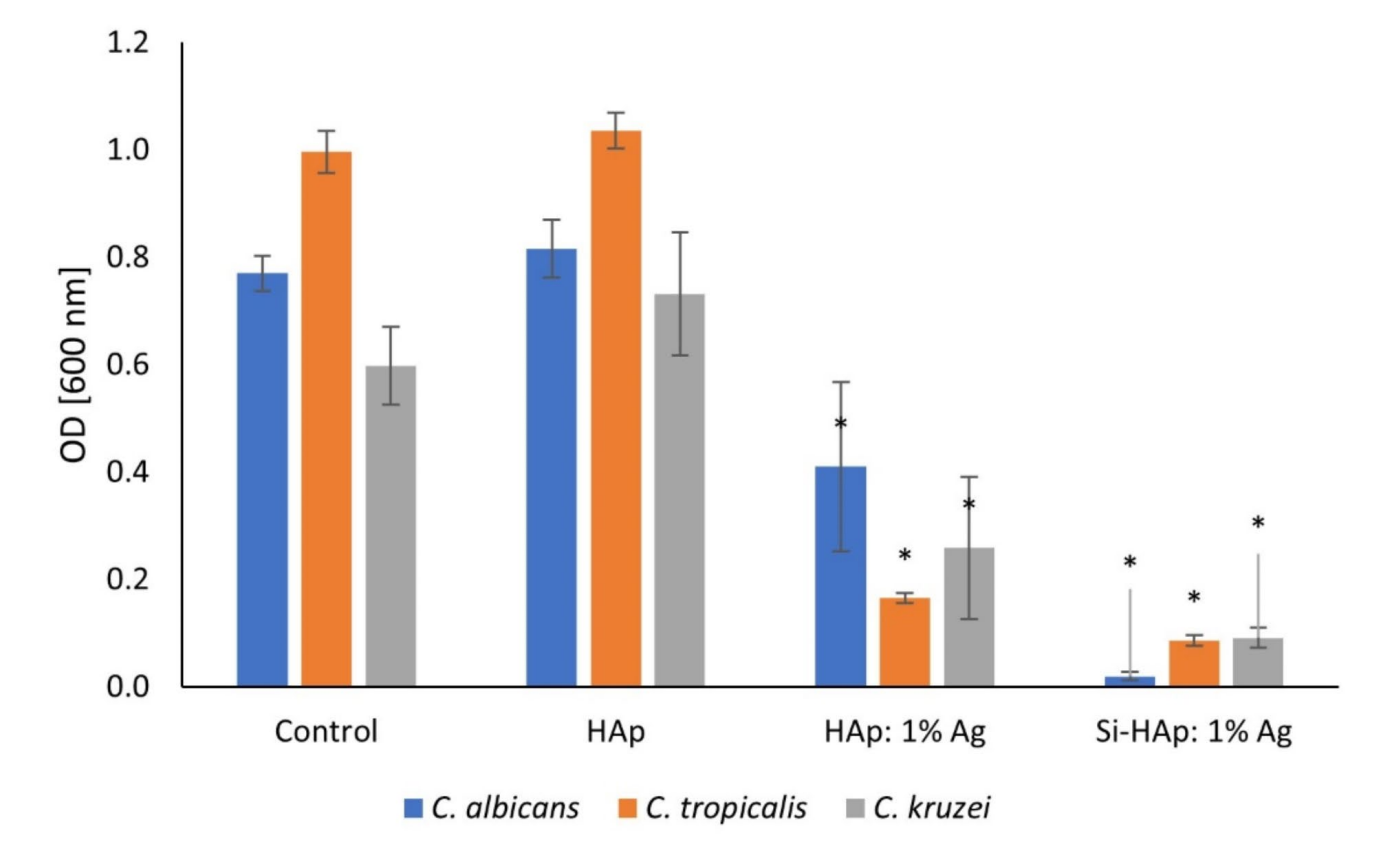
The inhibition of *Candida albicans*, *Candida kruzei* and *Candida tropicalis* growth by the control group, the undoped HAp, HAp: 1 mol% Ag^+^ and Si-HAp: 1 mol% Ag^+^ NPs, mean ± SD, *n* = 3. *- statistically significant (*p* < 0.05).

Antifungal activity of silver-doped nanoapatites was tested against three reference *Candida* sp. strains: *C. albicans*,* C. kruzei* and *C. tropicalis*, and the results are shown in Fig. 8. These chosen species are among the most common human pathogens and are associated with candidiasis^50^. Previously tested HAp substituted with 3 silica groups showed strong antifungal effects against multiple yeast-like strains^30^. Here, we compare the inhibitory effect of Ag^+^-doped HAp modified with silicate group and un-modified silver-doped HAp. We showed the stronger antifungal effect of silica-substitution, especially in the case of *C. albicans*, in which growth was inhibited almost completely. The difference in the inhibition of *C. tropicalis* and *C. kruzei* growth was lower but still statistically significant (*p* < 0.05). Silver, especially in the form of nanoparticles, may have multiple effects on fungal cells, including oxidative stress and membrane disruption that would lead to cell death^51^. Antifungal properties of hydroxyapatite are desired due to its application in dental implantation. *Candida sp*. present in oral cavity are common agents of implant infections as they can adhere to its surface and form biofilms^52^ Increasing resistance to antibiotics among *Candida sp*. generates the necessity of developing alternative methods to combat fungal infections^53^. Implant materials with incorporated antifungal ions are a good candidates to prevent the infection.

## Conclusions

This study elucidated the significant role of cationic and anionic doping in nanocrystalline hydroxyapatite with bioactive additives such as Ag^+^ ions and (SiO_4_)^4−^ groups. It was demonstrated that minimal silver doping (1 mol% of calcium) and anionic doping with less than one silicate group significantly influenced the antifungal properties of the synthesized materials. Utilizing a microwave-assisted hydrothermal technique followed by low-temperature sintering (450 ºC), we successfully obtained the crystalline phase of hydroxyapatite across all types and compositions of doped HAp nanosized particles.

Our findings revealed that silver and dual doping with silver and silicate groups substantially affect the morphology of hydroxyapatite nanocrystals. Undoped HAp nanocrystals exhibited an elongated rounded shape with a wide size distribution. Doping with 1 mol% Ag^+^ ions resulted in the formation of longer, more elongated particles with a narrower length distribution. Dual doping (Si-HAp: 1 mol% Ag^+^) further enhanced this effect, producing nanorods. Both HAp: Ag^+^ and Si-HAp: Ag^+^ demonstrated significant antibacterial activity against gram-positive and gram-negative bacteria, irrespective of the presence of the silicate group. However, regarding antifungal effect, the silicate groups enhanced the activity with it. High antimicrobial properties of studied hydroxyapatites make them promising medical materials that decrease a threat of an infection.

This study highlights that the antibacterial properties of doped hydroxyapatite are primarily attributable to silver ions, while the silicate group’s contribution (in synergy with silver ions) is most evident in antifungal activity. Given the wide-ranging medical applications of hydroxyapatite and the growing challenge of drug resistance among microorganisms, developing novel biologically active materials like those explored in this research is crucial for overcoming infections.

## Data Availability

The datasets analysed during the current study are available from the corresponding author on reasonable request.
